# Digital Reef Rugosity Estimates Coral Reef Habitat Complexity

**DOI:** 10.1371/journal.pone.0057386

**Published:** 2013-02-21

**Authors:** Phillip Dustan, Orla Doherty, Shinta Pardede

**Affiliations:** 1 Department of Biology, College of Charleston, Charleston, South Carolina, United States of America; 2 Coral Reef Projects, Biosphere Foundation, Big Pine, California, United States of America; 3 Indonesia Marine Program, Wildlife Conservation Society, Bogor, Jawa Barat, Indonesia; Leibniz Center for Tropical Marine Ecology, Germany

## Abstract

Ecological habitats with greater structural complexity contain more species due to increased niche diversity. This is especially apparent on coral reefs where individual coral colonies aggregate to give a reef its morphology, species zonation, and three dimensionality. Structural complexity is classically measured with a reef rugosity index, which is the ratio of a straight line transect to the distance a flexible chain of equal length travels when draped over the reef substrate; yet, other techniques from visual categories to remote sensing have been used to characterize structural complexity at scales from microhabitats to reefscapes. Reef-scale methods either lack quantitative precision or are too time consuming to be routinely practical, while remotely sensed indices are mismatched to the finer scale morphology of coral colonies and reef habitats. In this communication a new digital technique, Digital Reef Rugosity (DRR) is described which utilizes a self-contained water level gauge enabling a diver to quickly and accurately characterize rugosity with non-invasive millimeter scale measurements of coral reef surface height at decimeter intervals along meter scale transects. The precise measurements require very little post-processing and are easily imported into a spreadsheet for statistical analyses and modeling. To assess its applicability we investigated the relationship between DRR and fish community structure at four coral reef sites on Menjangan Island off the northwest corner of Bali, Indonesia and one on mainland Bali to the west of Menjangan Island; our findings show a positive relationship between DRR and fish diversity. Since structural complexity drives key ecological processes on coral reefs, we consider that DRR may become a useful quantitative community-level descriptor to characterize reef complexity.

## Introduction

Goreau's 1959 seminal paper on the zonation of West Indian corals highlighted hermatypic corals as the structural architects of the reef [1]. Influenced primarily by light, water movement, and sedimentation, he emphasized that individual growth forms aggregate to give the reef its morphology, including three dimensionality, and zonation. Robert MacArthur, examining the relationship between mobile animals (birds) and the more static structural community members (trees), demonstrated that more physically complex habitats supported higher bird species diversity [2]. Independent of MacArthur, Risk demonstrated a similar relationship between coral reef fish and substrate complexity which he termed rugosity, estimated as the ratio of the length of a straight line transect to that of a flexible chain draped over the reef along the same transect [3]. Since then, reef rugosity has been examined at scales ranging from centimeters to kilometers employing chain ratios [4], [5], visual categories [6], small-scale measurements [4], [7], acoustic backscatter [8], video image pixel brightness [9], [10], aerial-based lidar [11]–[13], and raster satellite imagery [14]. In most cases, a positive relationship has been found between fish community structure and structural complexity [3]–[17]. Rugosity has also become a metric to examine the structural changes that reefs undergo as the framework building corals die, rates of bioerosion overtake accretion, and the reefs eventually flatten [18].

Risk's original chain/tape ratio analog method was universally accepted because it was simple, inexpensive, and captured the essence of rugosity in a simple ratio. But chains tangle easily with reef organisms and their substrate and the ratio is an imprecise descriptor of structural complexity across a wide range of scales. Sampling techniques that increase small scale spatial resolution are time consuming [7], require multiple chains of varying link size [5] or intensive computer processing of video signals [9], [10], and are generally difficult to deploy at the scale of a typical 25–50 meter coral reef monitoring transect. The opposite mismatch in scale occurs with remote sensing as the pixel size of most readily available imagery equals or exceeds the finer scale indices of rugosity.

In this communication we describe a new technique to digitally parameterize coral reef structural complexity with a diver operated recording digital submersible level gauge designed to monitor groundwater water levels.

Preliminary data are presented suggesting a positive relationship between digital reef rugosity (DRR) and fish diversity across a number of different habitats on shallow water Balinese coral reefs, reinforcing the conclusion of others that structural complexity is a fundamental ecological property of reef communities [3], [4], [8], [9], [19]. We further show how the data gleaned in this study can be used to assess the topographical variability and scale of coral reef ecosystems.

### Study Site: Menjangan Island Coral Reef Ecosystem

**Figure 1:**
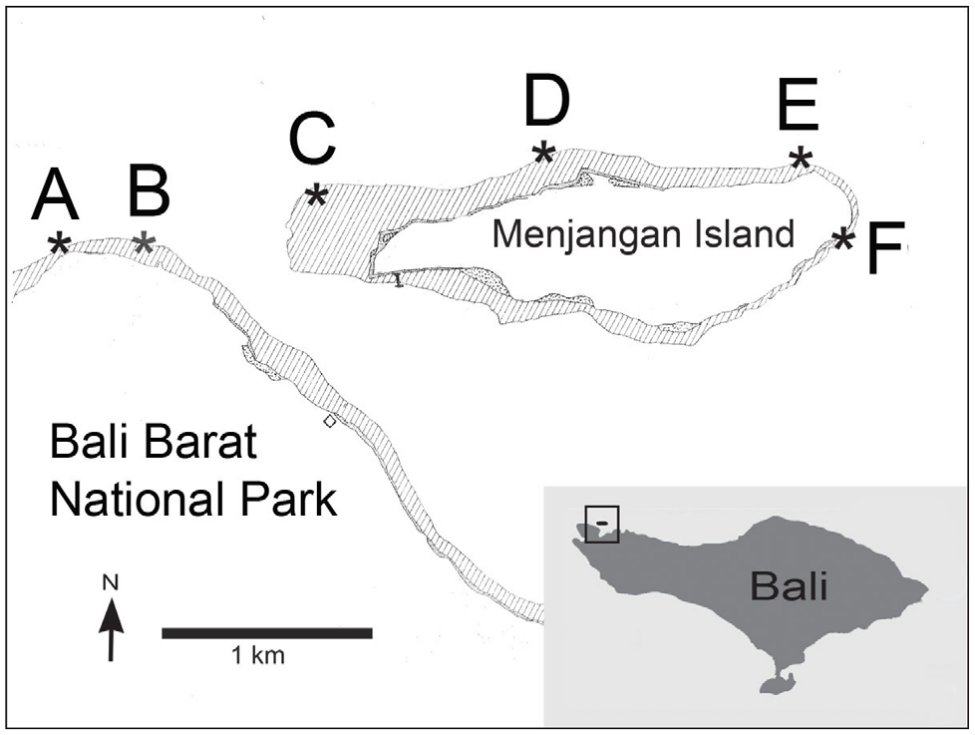
Digital Reef Rugosity study sites on the northwest corner of Bali, Indonesia. Study sites on mainland Bali at Kelor Point (A and B) and on Menjangan Island at Northwest Corner (C), Pasir Putih (D), Northeast Corner (E), and Pos 2 (F).

Four sites on Menjangan Island off the northwest corner of Bali, Indonesia (Northwest Corner, Pasir Putih, Northeast Corner and Pos 2) and one on mainland Bali to the west of Menjangan Island (Kelor Point), were surveyed (Figure 1). The reefs are dominated by monospecific stands of non-*Acropora* foliose and branching corals fitting into the r-K-s ternary classification CC = 2 of Endinger and Risk for Indonesian coral reef conservation [20]. Fifty-three of sixty-one known scleractinian genera and over one-hundred twenty-five species of reef fishes have been reported in this region of Bali [21], [22]. General reef morphology consists of a very shallow reef flat with a short drop to a terrace at 3–8 m that transitions into a forereef with a relatively steep, sometimes vertical, reef wall face beginning at 8–12 m [20]. The reef community is often luxuriant at the edge of the break in slope where small sill reefs develop along the tops of near vertical walls. In spite of Menjangan Island being within Bali Barat National Park the reefs have been degraded by blast fishing (reduced but ongoing, with impact craters observed as recently as 2012), overfishing even though fishing is allowed only for personal consumption, bleaching from elevated seawater temperature (1998, 2009 and 2010), severe crown-of-thorns starfish infestation (1997), ongoing anchor damage, and the chronic burden of plastic debris. The mainland site, Kelor Point, possessed large sections of rubble interspersed with stretches of virtually intact coral reef. The degraded areas were dubbed the “Killing Fields” because the destruction was near complete with no signs of coral recovery and/or recruitment observed on the mobile rubble substrate. All of these disturbances highlight the need for more assiduous ecosystem-based management [23].

## Methods

Our field studies were conducted in calm seas on the terrace between the reef flat and wall communities. At each site a 20–50 meter transect tape was set parallel to the general reef zonation (approximately perpendicular to swell direction) working along bathymetric contours within rather than across habitat zones. Transects varied in length depending on the patch size of reef substrate (healthy, rubble, etc) and depth (operational dive time).

Structural complexity was parameterized with fine scale pressure measurements recorded by a digital level gauge, an instrument normally used to track groundwater or stream levels (Onset Computer Company #U20_001-02, http://www.onsetcomp.com/products/data-loggers/u20-001-02). The ceramic pressure transducer of the instrument has a nominal operating depth range of 0–30 meters with a resolution of 0.41 cm and an accuracy of +/− 1.5 cm over its depth range. Temperature recording (0–40 °C, 12-bit resolution with ±0.37 °C accuracy) enables a diver to profile water column temperature and/or assess the fine scale distribution of temperature on the reef. The instrument has the capacity to record 42,400 individual data points at intervals as fine as one second, equaling almost 4 hours of data collection for pressure, temperature, time, and battery voltage which can be extended to almost 6 hours by omitting the battery voltage recording. The instrument communicates with a computer via an optically coupled base station and proprietary software (Onset Optical Base Station, Base-U-4). The level gauge can be programmed to start recording at a predetermined time which alleviates the necessity of bringing a computer into the field. Recording stops when the instrument's memory capacity is reached or by software control. Thus the instrument logs data continuously for an entire dive or day in the field until its memory is full. The actual useful data are subsets from the raw file after it has been downloaded and exported to a spreadsheet. While this greatly simplifies operation underwater, the operator must keep careful track of time and develop a protocol (as described in the next paragraph) to embed metadata information such as transect measurement start and stop into the constantly streaming raw data recording.

Digital rugosity transect data recording, at one-second intervals, began on the surface to estimate barometric pressure at sea level. Then the instrument was equilibrated at the sea surface to ensure an accurate temperature descent profile. DRR transects began by resting the instrument on the surface of the substrate at the transect starting point for 2–4 minutes to measure wave height variability. The beginning of a transect was marked by raising the probe vertically 0.6–0.8 m above the reef quickly one to three times to mark the data file with recognizable spikes. The diver then carefully and slowly swam along the transect line with the probe as close as possible to the reef contour without bumping the bottom. The probe was quickly raised 1–2 times at each five meter transect tape mark for distance calibration. The end of a transect was marked with 3–5 spikes and then resting the probe on the bottom for 1–2 minutes. The whole procedure took approximately 7–9 minutes for a twenty-five meter transect.

**Figure 2:**
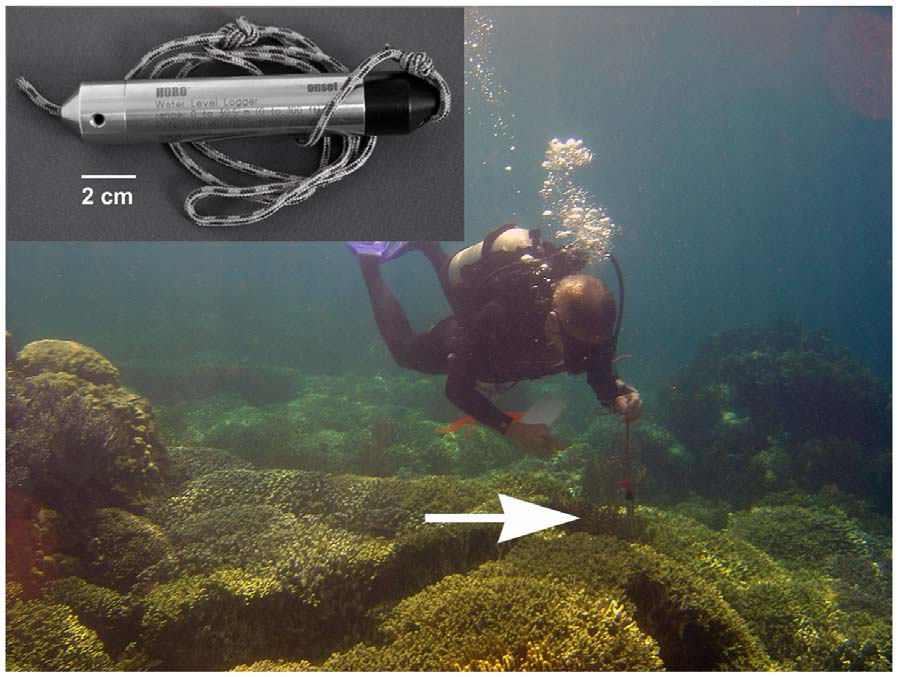
Diver demonstrates DRR methodology with inset showing level gauge. Digital Rugosity measurements are accomplished by using the instrument as a plumb-bob to slowly follow the contour of the reef as closely as possible. The pressure transducer is located near the end of the instrument closest to the substrate. The recorded pressure depth is offset from the actual reef substrate depth by the distance between the transducer and the substrate (photo A Alling).

An open reel 50 m fiberglass tape with metric graduations was used for a transect line because the centimeter scale tape markings helped guide swimming speed such that data sampling rate approximated 10 cm sec^−1^ along the taught linear tape. Suspending the probe from a short chord as though it were a plumb-bob facilitated following the bottom contour (Figure 2). It was easier to control the height of the instrument above the substrate and to regulate swimming speed and direction by swimming into the prevailing current. Very often the diver would need to swim diagonally across the transect line, crabbing into the current, to keep the level gauge on its path.

The raw level gauge data were downloaded, exported in ASCII format to a spreadsheet for atmospheric pressure correction and conversion into units of depth (meters), and parsed into individual transects. The distant marks were notated in the data file and used to examine the rate of travel (points-per-meter) for consistency. The contour of the reef along each transect was calculated by subtracting the deepest point from all other depths (relative depth). Consecutive depth differences were calculated from these data by subtracting each point from the next point [7]. We chose to characterize structural complexity as the standard deviation of the sensor output (DRR_STD_) as the standard deviation describes the variation of a set of measurements. This is different from the traditional rugosity chain/tape ratio but it has been widely used in many studies that do not employ the traditional methodology [8]–[13]. Additionally, DRR_STD_ can be calculated from raw sensor data because all the calibration factors are constants which do not affect transect variability. Fast Fourier transform (DRR_FFT_) was employed to explore the spatial distribution of structural complexity at horizontal scales within transects. The pressure measurements were subject to Single Series Fourier Analysis with an even number of samples based on the assumption of equally spaced sampling points at 10 cm intervals along each transect (STATISTICA, ver. 6. www.statsoft.com).

Benthic community percent cover (lumped stony corals, soft corals, and other functional groups) was estimated by pointcounting consecutive still digital photographs of each transect [24]. Fish populations were visually censused by divers along transects in general accordance with Wildlife Conservation Society protocol [25]. Fish abundance and species were recorded along a 2 m wide belt for small fish (<10 cm length) and a 5 m wide belt for larger fish (>10 cm length). Fish were identified according to the taxonomy of Lieske and Myers [26] and Randall et al. [27]. The field records of fish abundance and size were standardized to abundance hectare^−1^ and biomass hectare^−1^ using published length-weight relationships at four organization levels: species, genera, families and morphological groups [28]. Six transects on three reefs (Transect Numbers 1–5 & 7) were surveyed for fish, benthic community cover, and DRR. The other transects (Transect Numbers 6, 8–10) were censused for cover and DRR but without fish data (Table 1). All transects were conducted below the reef flat and above the drop off. Transects were censused once due to the brevity of our expedition. Non-parametric statistical analyses were used to be conservative as we could not be assured that our data (fish, coral, or DRR) were normally distributed.

**Table 1:** Study Sites.

Site*	Location	Reef Type	Coral Condition	Depth (m)	Transect Length (m)	Latitude (south)	Longitude (east)
KEL1-SC	Kelor Point	shallow terrace	good	3.5	23	08.09280°	114.48694°
KEL1-SR	Kelor Point	shallow terrace	rubble	4	30	08.09280°	114.48694°
KEL2-SC	Kelor Point	shallow terrace	good	3	23	08.09280°	114.48694°
KEL2-SR	Kelor Point	shallow terrace	rubble	4	30	08.09336°	114.49050°
PSR-SC	Pasir Putih	shallow terrace	good	4	48	08.09033°	114.51250°
PSR-DC	Pasir Putih	upper forereef	good	9	45	08.09033°	114.51250°
NWC-DC	Northwest Corner	upper forereef	good	9	50	08.09117°	114.49960°
NWC-SC	Northwest Corner	shallow reef	good	3	26	08.09117°	114.49960°
NEC-DC	Northeast Corner	shallow coral	good	7	23	08.09213°	114.52664°
POS2-DI	Pos 2	upper forereef	impacted	9	25	08.09577°	114.52829°

* Letters before the hyphen indicate the location, the S or D after the hyphen represents shallow (<5 m) or deep (>5 m) and the following C, R, or I represents coral, rubble or impacted zones.

## Results

### Benthic community

**Figure 3:**
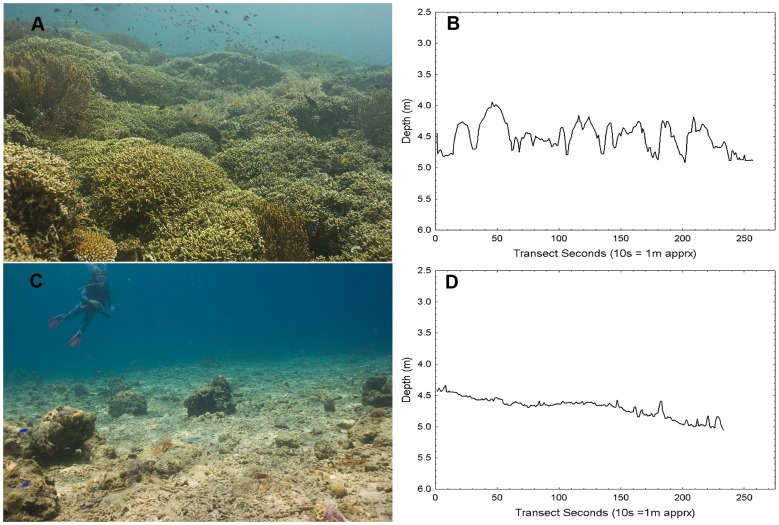
DRR transects in rich reef and adjacent rubble sites at Kelor Point. The fringing reef at Kelor Point alternates between rich undamaged coral reef (KEL2-SC (A and B)) and barren unstable rubble (KEL1-SR (C and D)). The peaks along the rich coral DRR transect (B) reflect the contribution coral colonies make to the canopy morphology. A comparison of the two DRR transects (B and D) illustrates the contribution live coral make to the reef canopy profile. The difference in depth between these two transects approximates the biovolume of the living reef. The transects are nearly equal in linear length, but the rubble transect contains fewer points because it took less time to swim (Table 5). (photos P Dustan).

The study sites spanned a wide range of combined stony and soft coral cover (Table 2). Coral reefs on Menjangan Island, a protected conservation area, ranged from 21 to 89 percent coral cover. At Kelor Point, we encountered large areas of rubble composed of branching coral fragments that were immediately adjacent to extremely luxuriant reef with 59–73 percent coral cover and no visible signs of impact (Figure 3). Algal cover (combined red, green, and alga turf) was generally below 5–7% even at sites with very low coral cover.

**Table 2:** Percent cover of functional groups.

Site	Stony Corals	Soft Corals	Macroalgae	Algal Turf	Red Crustose Algae	Porifera	Echinoids	Sand	Rubble	Other	n/c
KEL1-SC	59.4	1.7	0	0.9	2.3	1.1	0	5.1	29.4	0	0
KEL2-SC	73.7	0	0.3	1.9	5.2	2.7	0	0.3	15.9	0	0
KEL1-SR	1.6	0	0	0	1.3	0	0	15.9	81.2	0	0
KEL2-SR	1.4	0	0	0.5	1	1	0	5.2	91	0	0
PSR-SC	10.3	11.1	0	2	0.9	0.9	0	50.6	24.3	0	0
PSR-DC	41.2	9.4	0	1.1	4.7	5.8	1.8	0.7	31.1	0.4	4
NWC-SC	71.1	17.8	0	0.7	3.3	1.5	0	0	5.2	0.4	0
NWC-DC	44.3	3.6	0.4	5.7	1.6	6.6	0.2	0.5	35	2.1	0
NEC-DC	50.8	36.2	0.4	2.6	1.9	1.9	0	0.8	5.2	0.4	0
POS2-DI	1	1	0	0	0	2	0	2.5	93.5	0	0

### Digital Reef Rugosity

Ten transects varying in length from 23 to 50 meters with 6 of the surveys coinciding with fish counts were completed (Table 3). The sampling rate ranged from 6 to 13.37 samples/meter with a mean of 10.51 samples/meter and mode of 10 samples/meter. The two transects with the lowest sampling rate were both in areas of highly disturbed reef possessing little relief while transects with sampling rates higher than 12 samples/meter were in more complex communities. Stationary measurements recorded before and after transects were less than 1% of the vertical reef relief of most transects owing to calm seas and the fact that wave height became a smaller fraction of the signal as depth increased.

**Table 3:** Digital Reef Rugosity sampling statistics.

	KEL1-SC	KEL2-SC	KEL1-SR	KEL2-SR	PSR-SC	PSR-DC	NWC-SC	NWC-DC	NEC-DC	POS2-DI
Length (m)	23	30	23	30	48	45	26	50	23	25
Samples (n)	258	300	233	180	484	597	315	500	336	193
Samples/meter	11.2	10.0	10.1	6.0	10.1	13.3	12.1	10.0	14.6	7.7
Mean Relief	0.41	1.25	0.36	0.26	1.01	0.87	0.76	1.07	0.54	0.54
Max Relief	0.98	2.16	0.72	0.63	1.86	1.65	1.73	2.52	1.10	0.96
Std Dev (m)	0.22	0.39	0.16	0.16	0.43	0.41	0.32	0.68	0.25	0.23

**Figure 4:**
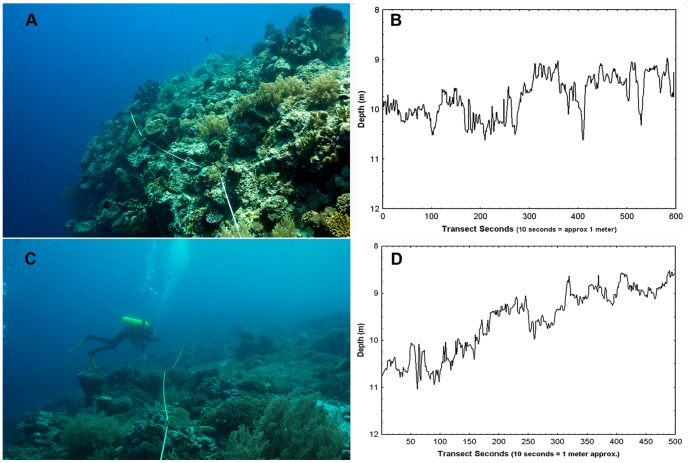
DRR transects in deeper reef zones. Pasir Putih (PSR-DC, (A and B)) and Northwest Corner (NWC-DC, (C and D)) were deeper and relatively undamaged sites on Menjangan Island with rich coral and fish communities. Both transects traverse along the top of a steep forereef slope. (photos P Dustan).

The richest of the reefs we sampled were virtually intact with bush-like branching colonies closely packed to form a dense, near-continuous canopy. Transects on the deeper forereef were more complex as the coral colonies of many different morphologies (branching, ramose, flattened, whorled, etc.) grow seaward and upwards, rather than simply upwards as in shallow water (Figure 4). DRR_STD_ varied between transects and reefs but was not significantly correlated with coral cover (Kendall tau = 0.2 ns). Levels of DRR_STD_ may be characteristic of a particular reef zone and/or morphology but the sample size was too small to make any assertions.

**Figure 5:**
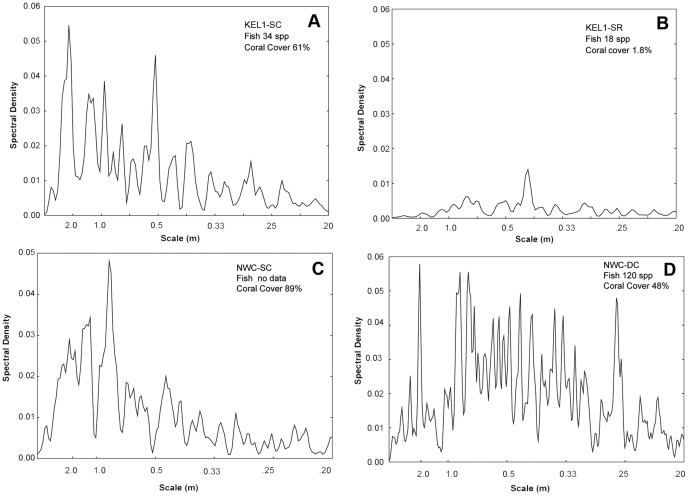
Scale of structural complexity revealed by Fast Fourier analysis of DRR. Spectral Density plots of DDR transects at Kelor Point (3–5 m) for a rich coral transect at KEL1-SC (A) and adjacent rubble site at KEL1-SR (B). Periodograms C and D characterize transects at Northwest Corner for rich coral cover in shallow (NWC-SC, 3 m) and deeper (NWC-DC, 9 m).

The periodograms resulting from Fast Fourier Transform varied between sites and depth. Highly degraded reef displayed very little variability across scale compared to an adjacent nearly intact rich shallow reef (Figure 5A and 5B). Richly covered shallow reefs with similar morphologies displayed similar periodograms with highest spectral densities of rugosity between 2–0.5 m scale (Figure 5A and 5C). The deeper region of the rich reef at Northwest Corner displayed a shift in the spectral density to a higher frequency (smaller scale) (Figure 5D).

### Fish Community Census

More than 120 species of fish were observed whose abundance, biomass, and diversity varied widely across sites (Table 4). These differences were most apparent by directly comparing the number of species within the trophic groups represented by five families at the rich coral sections of Kelor Point (KEL1-SC and KEL2-SC) with the adjacent damaged section (KEL1-SR) (Table 5). The rich coral area possessed a greater number of species in all families compared to the rubble sections, except for the Scaridae (parrotfish). There were no herbivorous Scaridae observed at the healthy reef sections, and yet, in adjacent sections of fine reef rubble a few meters away 3 species of parrotfish and only fish in two trophic guilds were observed.

**Table 4:** Fish census summary.

Site	Species (n)	Total Fish Counted (n)	Fish Abundance (no.ha-1)	Biomass (kg.ha-1)	H'_fish_
KEL1-SC	39	706	138440	492.3	0.90
KEL1-SR	20	193	36200	616.1	0.94
KEL2-SC	27	250	46280	746.0	1.08
PSR-SC	64	289	34240	1350.8	1.69
PDR-SC	90	370	62000	3022.6	2.59
NWC-DC	120	526	132960	2543.7	3.04

**Table 5:** Trophic structure of fish families at Kelor Point.

	Rich Coral Reef								Coral Rubble							
	KEL1-SC & KEL2-SC								KEL1-SR							
		Trophic Group*								Trophic Group*						
Family	Species (n)	h	d	co	ca	i	o	p	Species (n)	h	d	co	ca	i	o	p
Acanthuridae	2	1	1						2	1	1					
Chaetodontidae	2			2					0							
Labridae	13			1	3	7		2	0							
Pomacentridae	15						8	7	7						4	3
Scaridae	0								3	3						

* h = herbivore, d = detritivore, co = coralivore, ca = carnivore, i = benthic invertivore, o = omnivore, p = planktivore

**Figure 6:**
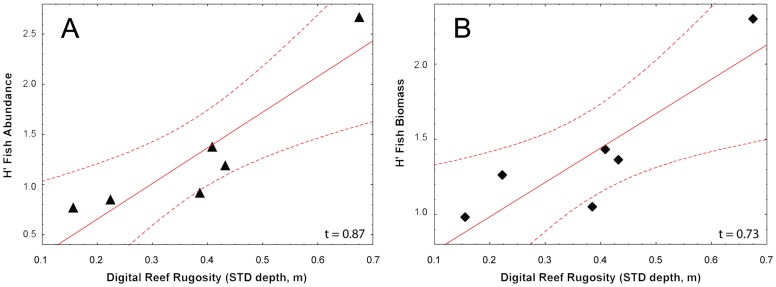
Fish biodiversity diversity correlated with reef structural complexity. Fish biodiversity based on the abundance of fish (A) or the calculated biomass of each species (B) is significantly correlated with DRR_std_ (Kendall tau τ = 0.87 and 0.73 respectively, p<.05 for both. The dashed lines delimit 95% confidence limits).

There was no significant correlation between fish density (abundance or biomass) and DRR_STD_ as has been seen in other studies of rugosity [3], [4], [6], [7], [9], [11], [13]–[17], [19]. However, there was a positive correlation between DRR_STD_ and fish species diversity (Shannon index (H′ = −∑ p_i_ Log p_i_) based on either fish species abundance or estimated biomass (Kendall tau = 0.87 and 0.73, p<.05, Figure 6).

## Discussion

In this communication we have presented a novel digital method to parameterize the structural complexity (rugosity) of coral reefs using a robust, off-the-shelf recording submersible pressure gauge. Initially, we have chosen to use the standard deviation of these fine scale pressure measurements as an estimate of rugosity but, with development, there may be other statistics that perform better. DRR measurements are relatively easy to carry out by a skilled SCUBA diver. The diver must swim the transect at a relatively constant swimming speed and use good buoyancy control; both are achievable with practice and concentration. Since it is a pressure measurement, waves and swell of any significant size will add information to the data. Increased wave height will add noise to the signal. Deeper transects will experience less “noise” from waves and/or swells as their signal becomes proportionally smaller with depth. At our sites below 5 meters the “noise” added to the variation in pressure was insignificant compared to the vertical relief of the coral reef. In some instances the use of a second stationary reference instrument may be helpful to remove wave variability in post processing, but one also has to recognize when it is too rough to work as it is far less complicated to confine data collection to calm sea conditions. Since diving conditions and substrates vary widely, each investigator will have to adapt the methodology to their specific study site conditions.

The Menjangan Island fringing reef community provided a range of reef habitats to explore the utility of DRR at scales commensurate with the scale of many coral reef monitoring programs (10 to 50 m transects). The positive correlation between DRR_std_ and fish diversity corroborates earlier work on fish species richness and reef structural heterogeneity analogous to the classic work of MacArthur for terrestrial forests [2]. With further refinement, the technique should provide for a more quantitative exploration of niche dimensionality on coral reefs. Curiously, there was no significant correlation between fish or coral population structure and the consecutive differences between points along a DRR transect. This measurement-to-measurement variation describes a finer scale variability of habitat complexity that is nested within the standard deviation [7]. Seemingly, fish population structure responds to the larger scale topography, rather than the fine scale variation of their habitats. Alternatively, more synoptic data collection may be needed to discern the effects of finer scale vertical and/or horizontal structural complexity.

It is also somewhat perplexing that there was no significant correlation between coral cover and DRR_std_ because stony corals and soft corals generate the topographical structure. High coral cover often generates a tightly knit canopy which reduces rugosity but may impart a DRR signature that will have diagnostic importance for long term monitoring as has been suggested for monitoring tropical forest systems with remote sensing [29]. At the Kelor Point Killing Fields, the stony and soft coral cover had been reduced to near zero and the area resembled a parking lot covered with branching coral fragment gravel. In adjacent nearly intact sections of reef, the close knit canopy was 0.5 to 2 meters above the substrate (Figure 3). The difference between these two comprises the biovolume of the reef, which might be considered as the third dimension of structural complexity. Without witnessing the destructive events it was not possible to determine the exact perturbation that created the Kelor Point ‘Killing Fields’, but fish bombing and/or coral mining that were once common in the area are prime suspects [22], [30]. Regardless of the cause, such sites as well as vessel groundings, anchor damage, blast fishing, and other anthropogenic disturbances could be surveyed and monitored efficiently with DRR. Developing an index incorporating DRR and biovolume would be useful for these long-term, permanently marked transect-scale monitoring and reef restoration projects.

Fast Fourier analysis partitioned the variation in structural complexity as a function of horizontal transect scale within the range of 0.2 m to approximately 5 m. The one-second sampling rate approximating 10 cm of horizontal distance enabled a spatial resolution of 20 cm following Nyquist sampling theory where the smallest scale detectable is twice the sampling interval [31]. For example, a continuous signal, such as music, can only be reconstructed when the Nyquist frequency exceeds the highest frequency of the signal. If one imagines the digital points of a reef rugosity transect as a continuous drape over the reef, the smallest frequency (horizontal scale) that can be described will be twice the sampling frequency, 2 seconds or approximately 20 cm of transect. Swimming slower would increase the horizontal resolution but a slower swimming rate may make straight-line navigation more difficult depending on swell, current, and other sea and/or reef conditions. Here again, each investigator will have to refine this aspect of the technique depending on skill and desired resolution.

The periodograms of DRR_FFT_ reveal the distribution of rugosity as a function of scale (horizontal distance). DRR_FFT_ spectral density plots from two rich shallow reefs (Kelor Point and Northwest Corner) displayed strong variation at similar scales ranging from 0.5–2 m (Figure 5A and 5C). These reefs have similar coral canopies composed of tightly packed branching colonies approximately 0.5–1 m in diameter (Figure 2 and 3A). The striking contrast between a nearly intact shallow water reef with rich coral and an adjacent destroyed site (Figure 5A and 5B) illustrates the contribution coral colonies make to overall reef dimensionality. The deeper reef zone at Northwest Corner, had a spectral density plot that was more variable and dispersed across a broader scale (Figure 5D). Colonies in these deeper zones tend to be smaller and exhibit a wider range of morphologies with each apparently influencing reef rugosity differently [5]. The periodogram of NW Corner may also reflect the presence of narrow sand channels that are absent on the shallow reefs. While these data are preliminary, they suggest the spectral domain of DRR_FFT_ may improve our understanding of the contribution made by individual colonies to community structural complexity and reef morphology. Additionally, since fish and other mobile organisms tend to scale with their habitat [5], [19], DRR_FFT_ may provide further insight into the influence of habitat complexity on substrate preference and niche partitioning.

Digital Reef Rugosity can be applied across a wide range of spatial and temporal scales without sacrificing accuracy or precision since the length of a transect is virtually unlimited. Thus the technique provides a precise link between marine ecology and remote sensing which has eluded satellite-based remote sensing efforts because the smallest scale detectable from space is usually greater than the scale used in most ecological studies [5]. Additionally, finer-scale aircraft-based remote sensing might benefit from the increased resolution that DRR provides over the traditional chain methodology [11]. At yet smaller scales, measuring habitat complexity across different ecological zones and different environmental conditions could yield insight into the relationship between ecological zonation, hydrodynamics, and reef structure. For example, DRR_FFT_ analysis could be employed to characterize the relationship of reef canopies with prevailing seas or calculate hydrodynamic forces through an analysis of wave height time series measurements along depth transects using stationary instruments [32].

Globally, coral reef decline is embodied by the loss of coral cover, which is itself a proxy for reduced structural complexity analogous to terrestrial deforestation. Rugosity that is generated by coral colonies integrates ecological integrity, complexity, and vitality because these functions are biologically inseparable from structure on reefs. At some point in the degradation process the structural complexity of the reef ceases to support its fish community. In this time of dwindling management resources and accelerating reef degradation, a simple field technique that could help detect, or even predict, such tipping points would be an invaluable tool to help triage reefs for the most appropriate course of conservation; no-take policies might be applicable for overfished reefs but others might require rebuilding the physical structure to augment rugosity. Digital Reef Rugosity provides a modern method to more fully parameterize the fundamental community property of coral reefs so elegantly conceived by Risk over 40 years ago [3].
